# Cycle Syncing and TikTok’s Digital Landscape: A Reasoned Action Elicitation Through a Critical Feminist Lens

**DOI:** 10.1177/10497323241297683

**Published:** 2024-11-22

**Authors:** Emily J. Pfender, Katelynn L. Kuijpers, Claire V. Wanzer, Amy Bleakley

**Affiliations:** 1 Perelman School of Medicine, 6572 University of Pennsylvania, Philadelphia, PA, USA; 2 Department of Communication, 5972 University of Delaware, Newark, DE, USA

**Keywords:** cycle syncing, social media, critical feminist theory, reasoned action approach, menstrual health

## Abstract

Cycle syncing is a menstrual health trend on TikTok that involves aligning exercise and diet with the four menstrual cycle phases. Cycle syncing is part of the conversation on social media about women’s reproductive health. However, clinical research on the effects of cycle syncing is inconclusive, and there is the potential that this trend could further perpetuate misinformation and gender stereotypes. Research suggests that social media can affect health behaviors, highlighting the need to understand if women intend to participate in cycle syncing. Guided by the Reasoned Action Approach, this study used focus groups (*n* = 39) to examine young women’s attitudes, normative beliefs, and control beliefs about participating in cycle syncing, and critical feminist theory to sensitize resulting themes. Results suggest that normative beliefs emphasize support for the behavior among women, yet participants suggest that men would not support this behavior. Additionally, positive beliefs about cycle syncing content sourced from inconclusive scientific literature underscores concerns regarding the potential dissemination of misinformation in women’s health practices on social media. Findings also fit into a larger discussion about “hormonophobia” and contraception on social media. Theoretical implications for mixed methods research and future directions are discussed.

## Introduction

Social media platforms such as TikTok have emerged as powerful outlets for advocacy and information dissemination regarding women’s reproductive health and well-being. Cycle syncing has gained traction on various platforms as a popular health behavior. Cycle syncing involves aligning daily activities, including diet and exercise, with the four phases of the menstrual cycle (Krupp, 2023). Popular press articles and content creators suggest that the four phases of the menstrual cycle include the menstrual phase (i.e., days 0–7, when individuals have their period), follicular phase (i.e., days 8–13, when estrogen rises), ovulation phase (i.e., days 14–15, when ovaries release an egg), and luteal phase (i.e., days 16–28, when the uterus prepares to receive a fertilized egg) (Cleveland Clinic, 2023; Pfender, Wanzer, et al., 2024). Although the origins of cycle syncing date back to at least 2014 (Cleveland Clinic, 2023), recent social media trends have likely increased its popularity with some audiences, including professional athletes such as the U.S. women’s soccer team in 2019 (Rudnesky, 2023). On TikTok, the hashtag #cyclesyncing garnered 285M views in 2023 and was most popular in the United States, Canada, the United Kingdom, and Australia (TikTok, 2023).

In the United States, cycle syncing is focused on adjusting diet and exercise around the differences in mood and energy throughout the four phases of the menstrual cycle (Cleveland Clinic, 2023). For instance, the menstrual phase calls for lighter exercises like yoga, walking, and stretching, while the follicular phase recommends high-intensity workouts such as weightlifting (Rudnesky, 2023). Beyond exercise, cycle syncing also applies to dietary choices throughout the menstrual cycle. For example, nutrient-rich foods high in iron, vitamin C, vitamin K, and omega-3 fatty acids are suggested during the menstrual phase (Cleveland Clinic, 2023). These patterns in exercise and diet are thought to help alleviate some of the symptoms associated with menstrual cycles and allow one to be more in touch with their monthly hormone changes (Cleveland Clinic, 2023). Despite these claims, there is no previous research on the benefits or outcomes of cycle syncing. However, there is a wide body of research examining exercise performance and nutrition during the phases of the menstrual cycle, which lacks clinical consensus (Colenso-Semple et al., 2023; Rael et al., 2021; Sakamaki-Sunaga et al., 2016; Sung et al., 2014; Wikström-Frisén et al., 2017). TikTok content creators are therefore discussing the emerging trend of cycle syncing in the context of older research about menstrual cycle phases (Reis et al., 1995).

There is a lack of consensus regarding the effect of the menstrual cycle phases on physical exercise and performance-related outcomes. While female exercisers report that their menstrual cycle affects their exercise performance (Solli et al., 2020), many studies find no significant differences by menstrual phase in muscular strength or endurance (Colenso-Semple et al., 2023; Rael et al., 2021; Sakamaki-Sunaga et al., 2016). In a narrative review, Carmichael et al. (2021) report that “studies examining objective performance (using anaerobic, aerobic or strength-related tests) do not report clear, consistent effects on the impact of menstrual cycle on physical performance” (p. 1667). Other studies find that menstrual cycle phase affects women’s exercise performance (Sung et al., 2014; Wikström-Frisén et al., 2017). For example, some research finds that follicular phase–based strength training results in a greater effect on muscle strength than luteal phase–based strength training (Wikström-Frisén et al., 2017). Scholars argue that there is disagreement across the literature, likely due to flawed and incongruent methodological practices (Colenso-Semple et al., 2023; Pereira et al., 2020), and some sources disagree on how many menstrual phases exist (Carmichael et al., 2021; Cleveland Clinic, 2023).

Proponents of cycle syncing also encourage women to take more rest time during their menstrual phase and even “say no to work deadlines during low-energy phases” (Gupta, 2023). However, research suggests that the practice of cycle syncing in the context of exercise could be disadvantageous for female athletes as refraining from intense play during specific menstrual cycle phases could have negative financial and social implications (Pfender, Wanzer, et al., 2024). Even when significant differences in menstrual cycle phase and exercise performance are found, “the magnitude and direction of the effects are inconclusive” (Meingnie al., 2021, p. 1). The inconsistency of scientific findings underscores the challenge of definitively linking menstrual cycle phases to exercise performance, creating fertile grounds for TikTok content creators to present cycle syncing information in a piecemeal format.

Research on cycle syncing and diet is also inconclusive, and studies on total energy, micronutrient, and macronutrient intake during phases of the menstrual cycle demonstrate inconsistent or scant findings (Gorczyca et al., 2016). Few studies address nutrition and the menstrual cycle, and those that do have been criticized for lacking generalizability based on limited lifestyle considerations in participant samples, which rarely account for athletic status (Helm et al., 2021), exercise habits, or personal diet (Rogan & Black, 2022). Additionally, studies on diet and the menstrual cycle struggle to capture acute hormonal differences within individual cycle phases, due to the disparities in defining each phase’s timing (Rogan & Black, 2022). The lack of scientific consensus regarding exercise, nutrition, and the menstrual cycle may result in misrepresented recommendations on TikTok. Yet, TikTok content creators seem to believe cycle syncing can offer several health benefits including reduced menstrual symptoms, balanced hormones, and reduced acne (Pfender, Wanzer, et al., 2024).

The current research integrates critical feminist theory (CFT) with a health behavior theory—the Reasoned Action Approach—to provide a lens through which to analyze power dynamics and societal expectations associated with cycle syncing. Furthermore, this study seeks to understand women’s beliefs about cycle syncing, what factors might contribute to women choosing to practice cycle syncing, and how they see it fitting into their lives.

### Critical Feminist Theory

CFT emerged from critical race theory to address diverse forms of gender oppression (MacKinnon, 1983, 1991). The theory’s core assumptions highlight that gender oppression is normative, deeply rooted in society, and difficult to detect, urging us to challenge traditional gender ideals (MacKinnon, 1983). Consequently, the theory focuses on the importance of women’s experiential knowledge, emphasizing that unique voices and narratives must be considered within the framework of intersectionality (Wigginton & Lafrance, 2019), a term coined by Kimberlé Crenshaw to draw attention to disadvantages faced by women because of their race *and* gender (Crenshaw, 1991, 1997). The theory has been used to understand issues ranging from rape (Geisinger, 2011) to female athletes’ experiences (Villalon & Weiller-Abels, 2018), and motherhood (Ogunwole et al., 2023). By drawing from interdisciplinary frameworks and using platforms to advocate for social justice, proponents of CFT use it to understand systems of oppression and power.

CFT is particularly well-suited for studying cycle syncing. The theory’s recognition of gender inequities aligns with concerns that the practice of cycle syncing perpetuates stereotypes, biases, and inequalities related to menstrual cycles. For example, resting and refraining from exercise during the menstrual cycle could fuel gender inequities in athletics or professional environments. Additionally, it’s important to consider how class and socioeconomic status influence access to the resources necessary for cycle syncing, such as the ability to rest or modify schedules, which may be available to some but not others. For instance, a woman working a low-wage job with strict shift schedules may not have the flexibility to rest or adjust her workload based on her menstrual cycle. She might also lack access to nutritious foods or supplements that support cycle syncing due to financial constraints. In contrast, a woman in a higher-paying job with more control over her work hours may be able to modify her schedule to align with her cycle, take time off for self-care, and afford resources like fitness classes or specialized dietary plans that promote cycle syncing.

On the other hand, the theory sheds light on how the cycle syncing trend can be seen as a form of resistance to traditional gender norms, medicalization, and consumerism by promoting self-acceptance, collective knowledge, and solidarity among women. There is a community that has emerged around that practice of cycle syncing that often involves sharing experiences, advice, and knowledge among women on social media (Rudnesky, 2023). CFT emphasizes the power of collective knowledge and solidarity in resisting oppressive structures (Wigginton & Lafrance, 2019). Women suggest that cycle syncing has many health benefits and can reduce symptoms associated with menstruation (Pfender, Wanzer, et al., 2024). This knowledge can be empowering especially because female reproductive health and pain has been ignored throughout history (Amaro et al., 2001). Solidarity surrounding cycle syncing might be strengthened by salient female relationships, since recollections of menstruation are often associated with mother–daughter communication (Costos et al., 2002). By sharing information about cycle syncing, women can help each other navigate the challenges of their menstrual cycles, and it has the potential to emerge as an empowering and supportive practice for women in challenging traditional norms.

### Reasoned Action Approach

The reasoned action approach is used to examine and predict health behaviors (Fishbein & Ajzen, 2010) and suggests that intentions are the primary predictor of behavior. This approach also focuses on intention formation by asserting that attitudes, normative pressure, and perceived behavioral control are the three predictors of intention (Fishbein & Ajzen, 2010). Attitudes are conceptualized as the positive or negative evaluation of the behavior. They have an underlying set of behavioral beliefs. Norms are social pressures to engage in a behavior and include injunctive and descriptive norms. Injunctive norms are perceptions about what others think one should do, while descriptive norms are perceptions of what one thinks others are doing. According to the theory, important individuals that make up injunctive and descriptive norms are called “referents” (Fishbein & Ajzen, 2010). Perceived behavioral control is composed of autonomy and capacity. Autonomy refers to the extent to which individuals perceive themselves as having control over their actions and choices, and capacity refers to an individual’s perceived ability or resources to perform a certain behavior. Autonomy and capacity are made up of beliefs related to personal and external factors that act as barriers or facilitators to engaging in the behavior. Each of the three primary predictors of intention has a set of underlying salient beliefs.

The reasoned action approach provides a useful framework for examining beliefs about cycle syncing, as it has been used to understand menstrual cycles and reproductive behavior (Fisher & Fisher, 1998; Norouzi et al., 2018), and can identify key determinants around decision-making with regard to cycle syncing. Health content on social media has the power to influence attitudes toward health behaviors, including exercise and cervical screening appointments (Durau et al., 2022; Fielden & Holch, 2022). Furthermore, social media shapes cultural perceptions of norms through algorithmic social-learning processes (Brady & Crockett, 2024) so that individuals who engage with a post about cycle syncing will then receive more content about cycle syncing. More exposure to the same content on social media might give users the impression that the behavior is more socially acceptable and increase normative pressure (Hawkins et al., 2020). Additionally, content creators are interactive on social media and have been found to provide encouragement to their followers (Sokolova & Perez, 2021). Receiving encouragement about performing health behaviors may increase followers’ self-efficacy (Sokolova & Perez, 2021). These are only some of the ways attitudes, norms, and control may play a role in shaping individuals’ intentions to engage in cycle syncing after exposure to social media content.

Elicitation research is used when there is little known about the beliefs associated with a behavior, such as a new health phenomenon on TikTok like cycle syncing. Previous elicitation studies (Bleakley et al., 2022; Fishbein & Ajzen, 2010; Pfender & Bleakley, 2024) demonstrate a need to first examine underlying beliefs before further investigating new health behaviors. Cycle syncing is a newer health behavior that lacks clinical scientific consensus, making it an issue that is susceptible to health misinformation. Therefore, focus groups were used to elicit individuals’ attitudes, norms, and control surrounding cycle syncing using the following research questions:

RQ1 What are young women’s attitudinal beliefs about cycle syncing?

RQ2 Who are important referents when it comes to cycle syncing?

RQ3 What barriers and facilitators do young women mention when considering cycle syncing?

## Methods

Four focus groups were conducted on Zoom in October 2023 (*n* = 39). Participants were undergraduate students at a Mid-Atlantic University ranging in age from 18 to 22 (*M* = 19.74, *SD* = 1.13). Most participants identified as women (*n* = 38, 97.4%), with one participant identifying as non-binary. About 75% (*n* = 29, 74.4%) of participants identified as White and about 25% identified as Latinx (*n* = 10, 25.6%). The sample was largely heterosexual (*n* = 31, 79.4%) and single (*n* = 30, 76.9%). Demographic characteristics of the participants can be found in Table 1.

**Table 1.** Demographics.

	%	*n*
Gender		
Woman	97.4	38
Non-binary	2.6	1
Ethnicity		
Non-Hispanic/Latinx	74.4	29
Hispanic/Latinx decent	25.6	10
Race		
White	74.4	29
Hispanic/Latinx	15.4	6
Black	7.7	3
American Indian	2.6	1
Asian	2.6	1
Sexual orientation		
Heterosexual	79.5	31
Bisexual	10.3	4
Pansexual	7.7	3
Asexual	2.6	1
Relationship status		
Single	76.9	30
In relationship	23.1	9

*Note. N* = 39. The mean age was 19.74 years (*SD* = 1.13).

About half of the sample reported using birth control (*n* = 22, 56.4%) with one-third of participants identifying the birth control pill as their primary method (*n* = 13, 33.3%). Participants reported experiencing a variety of reproductive health conditions including menstrual pain (*n* = 18, 46.2%), amenorrhea (*n* = 4, 10.3%), and vaginismus (*n* = 2, 5.1%). More than half of the participants previously sought out information about cycle syncing on social media (58.9%, *n* = 23) and 71.8% reported having previously seen posts about cycle syncing on social media (*n* = 28). Further information about participants’ reproductive health can be found in Table 2.

**Table 2.** Descriptive Statistics for Birth Control and Reproductive Health.

	%	*n*
Use any birth control	56.4	22
Primary birth control		
Pill	33.3	13
Condoms	15.4	6
Hormonal IUD	12.8	5
Implant	7.7	3
Ring	7.7	3
Cycle tracking	5.1	2
Pull out method	5.1	2
Copper IUD	2.6	1
Reproductive health conditions		
Menstrual pain	46.2	18
Amenorrhea	10.3	4
Vaginismus	5.1	2
PCOS	2.6	1
Pelvic floor dysfunction	2.6	1
Premenstrual dysphoric disorder	2.6	1

*Note. N* = 39.

Participants entered the video conference, were welcomed, and were instructed to complete the written informed consent and demographics survey via a link in the chat. Once all participants completed the consent and survey, researchers discussed community guidelines. Researchers reminded participants that anything discussed in the focus group should not be shared and that there were no right or wrong answers, encouraging participants to speak freely. Participants were also welcomed to disagree with each other but were instructed to do so in a respectful manner. Following community guidelines, the researchers asked participants if they could define cycle syncing. After that, they showed participants a brief PowerPoint that explained the four menstrual cycle phases. The researchers used the PowerPoint to inform their explanation of cycle syncing, including recommendations for diet and exercise during each phase. The researchers designed the PowerPoint and cycle syncing explanation using previous content analysis research to depict trending and current social media content more accurately (Pfender, Wanzer, et al., 2024).

Next, researchers guided participants through a warmup exercise. The warmup activity was designed to help participants feel comfortable speaking up in the focus group. Participants were asked a variety of questions, including what type of social media they use and if they use social media to seek out health-related information. Participants were also asked if they follow any special diets, exercise, or self-care routines while on their periods. Finally, participants were asked about their familiarity with the concept of cycle syncing.

Following the warmup questions, the researchers asked a series of structured open-ended RAA questions to elicit beliefs (Fishbein & Ajzen, 2010). Cycle syncing attitudes were assessed by asking participants what good things or bad things could happen if they participated in cycle syncing. To capture normative pressure, participants were asked to list referents that would approve or disapprove of cycle syncing. Referents describe important people in a participant’s life. Finally, participants were asked to identify things that would make it easy or difficult to cycle sync to capture perceived behavioral control. Measures from the interview guide can be found in Table 3. The study received approval from the institutional review board at the sponsoring institution (IRB approval #: 2105426-2).

**Table 3.** Focus Group Measures.

Construct	Measure
Attitudes (expectancies)	What are some of the good things you think could happen if you cycle sync?
	What are some of the bad things you think could happen if you cycle sync?
Normative pressure (normative referents)	Who do you think are the people in your life that think you *should* cycle sync?
	Are there any people in your life who think you *shouldn’t* cycle sync?
Perceived behavioral control/self-efficacy (barriers and facilitators)	What are some things that might make it easy to cycle sync?
	What are some things that might make it difficult to cycle sync?

### Thematic Analysis

Braun and Clarke’s (2006) thematic analysis was used to analyze the focus group data. Thematic analysis is a flexible qualitative data analysis method which allows researchers to identify and define themes and patterns within a dataset and yields rich descriptive results. Braun and Clarke (2006) outline six steps for thematic analysis, which include “familiarizing oneself with the data, generating codes, searching for themes, reviewing themes, defining themes, and producing a report” (p. 87). The present study employed thematic analysis, sensitized by the core tenets of CFT (MacKinnon, 1983, 1991; Wigginton & Lafrance, 2019).

As outlined by Braun and Clarke (2006), researchers first reviewed the transcripts until saturation had been reached (Glaser & Strauss, 1967). Throughout the review of transcripts, researchers took descriptive notes and completed in-process memos (Emerson et al., 1995) which identify and elaborate upon preliminary analytic and theoretical contributions of field notes. Next, the three researchers individually generated initial codes using holistic coding (Saldaña, 2009) based on notable or prevalent responses throughout the dataset and analytic memos. Saldaña (2009) suggests that holistic coding is appropriate “when the researcher already has a general idea of what to investigate in the data” (p. 119), which was driven by the core tenets of CFT. As a group, the three researchers discussed their independently generated initial codes. Initial codes were collapsed and shaped to generate preliminary themes. The resulting themes were reviewed in relation to the dataset, to check their fit. Final themes were found to accurately describe patterns across the dataset, within the ideas and tenets of CFT. Finally, themes were defined and assigned an exemplar.

## Results

Several themes emerged for each of the Reasoned Action constructs, including attitudinal, normative, and control beliefs, totaling nine themes. For attitudinal beliefs, four themes emerged, including two themes pertaining to cycle syncing advantages and two themes pertaining to cycle syncing disadvantages. Next, we present three themes pertaining to normative beliefs. For each of the three normative belief themes, we reveal the specific individuals or referents who enforce normative pressure (e.g., mother, father, and sister). Finally, we present two themes for perceived behavioral control, including one theme specifying facilitators to cycle syncing and one theme specifying barriers to cycle syncing. Among the facilitators, one example emerged and among the barriers, four examples emerged.

### RQ1: What Are Young Women’s Attitudinal Beliefs About Cycle Syncing?

Participants mentioned several attitudinal beliefs (i.e., advantages and disadvantages) regarding cycle syncing. Four themes emerged: body positivity and self-acceptance, resistance to medicalization, cycle syncing is a one-size-fits-all approach to health, and cycle syncing practices can lead to internalized shame. For each of these four themes, we specify advantages and disadvantages.

#### Theme: Cycle Syncing Can Lead to Body Positivity and Self-Acceptance

##### Advantage: Self-Acceptance

Participants suggested that participating in cycle syncing would help them better understand their bodies. They felt that living in alignment with their menstrual phases would allow them to be more in tune with their bodies and anticipate what their bodies needed on any given day. For many participants, acknowledging the physical and emotional changes associated with the menstrual cycle meant avoiding negative self-perceptions:

I think it gives an opportunity, or like a capacity for grace with yourself. I know it [menstrual cycles] can also cause like weight gain and things like that. So maybe it’s like, you’re not necessarily thinking you suck at lifting, or you’re just getting worse at life, or you’re gaining all this weight like maybe it’s like … there’s a lot going on inside of me.

Many felt that cycle syncing could reduce pressure to always be perfect and practice more self-compassion: “Maybe like less frustration with myself. Like if I understood … why I was feeling the way I was because of what phase I was in.”

Other participants expressed that cycle syncing could help them better understand their bodies:

It really shows us the cycle that our body already wants to be in … I guess we would be more in touch with our body and we know, like, I’m feeling tired today, I should do this, this and this, but I’m feeling tired because of where I am in my cycle. So I feel we’d get closer with our body and better understand how and why we’re feeling a certain way.

#### Theme: Cycle Syncing Involves Resistance to Medicalization

##### Advantage: Symptom Relief

Rather than relying on traditional medical practices, participants described cycle syncing as an opportunity to naturally enhance reproductive, physical, and mental well-being. Participants mentioned several benefits, ranging from experiencing less menstrual pain and side effects to regulating one’s cycle, improving energy, and boosting mental health:

It could just boost your mood all around like make you just like more well-rounded and … improve your sleep… and boost your mood … just make you healthier all around.

Participants highlighted cycle syncing’s focus on healthy foods and regular exercise practices. They suggested that healthy eating and regular exercise can benefit reproductive health by reducing negative effects related to menstruation:

It might lessen some of your symptoms that you have during your period, if you get like bad cramps, or something like that. Eating better foods that are better for you and exercising might be able to lessen those effects.

When I’m more active, or I’m like eating better, I have more energy. So I feel like doing that, especially at a time when, like, you’re like menstruating, it’s like better for you. And it’ll … help it all around.

In the context of cycle syncing, medicalization may occur if natural variations in a woman’s menstrual cycle and associated changes in mood or energy are pathologized, overly medicalized, or stigmatized. Medicalization means taking what is normal and treating it as though it needs medical intervention or is inherently problematic. Yet, many participants viewed cycle syncing as a more “holistic and functional perspective to health” that could help them improve their well-being naturally.

#### Theme: Cycle Syncing Is a One-Size-Fits-All Approach to Health

##### Disadvantage: Untailored Health Advice

Participants raised concerns that cycle syncing practices lack tailored advice for individual menstrual cycles. They highlighted variations in women’s menstrual cycles and cautioned against a one-size-fits-all approach to cycle syncing.

It seems like a one-size-fits-all thing when women interact with their hormones differently than other women. For example, I will often time skip my period just by using my birth control method on purpose. So then I don’t follow the same kind of week by week that a lot of other women do and during my period I find that I enjoy doing like more intensive workouts than just yoga, because my cramps are really bad, and exercise really helps with that.

Similarly, others shared concerns that influencers might commodify cycle syncing by turning the practice into a marketable trend, potentially promoting standardized reproductive health plans, overlooking the diverse and individual nature of women’s experiences with their menstrual cycles.

If it gets … commodified and if influences are like, here is the period diet plan, it’s not going to be a one-size-fits-all because everyone’s period is different. Everybody’s body chemistry is different. So what might work for one person might not work for you.

#### Theme: Cycle Syncing Practices Can Lead to Internalized Shame

##### Disadvantage: Worsened Mental Health

Participants expressed concern about the adoption of extreme and unhealthy diet and exercise routines, leading to physical and emotional difficulties.

I think that it could potentially be harmful to younger girls if they’re taking it to an extreme possibly. For me, I think … I would know what’s healthy dieting and what’s healthy exercising but not everybody does know that and so that they might, kind of take it to the extreme and then be in a routine of unhealthy practices that would not only make, you know, everything worse for them, but then also make their period possibly harder for them.

Some participants worried that extreme diet and exercise could result in poor mental health:

It could also turn into a mental health problem because individuals who experience periods could internalize why these methods projected on social media aren’t working for them, and they could ultimately train their brain to think that I’m at fault within this system … when in reality it’s the system that’s failing the person. So I feel like the internalization is dangerous here.

Participants suggested that poor mental health because of cycle syncing could be driven by shame cycles, emphasizing the importance of intuitive and body-focused approaches rather than strict adherence to external standards during menstruation.

I … think it could lead to like a shame cycle in young girls of like indulging themselves on their period. Cause like, as we said, a lot of us do crave like sweets and like you know fast food and like young girls are really susceptible to like oh, well this thing on TikTok said that I should eat super healthy or I should eat soup. So now like I feel guilty if I eat XYZ or if I don’t go exercise.

### RQ2: What Do Important Referents Think About Young Women Cycle Syncing?

The second research question sought to understand important referents for young women’s normative beliefs about cycle syncing. Three themes emerged, including cycle syncing is influenced by a patriarchal system, generational differences for engaging in cycle syncing, and female solidarity in the practices that involve reproductive care. Specific referents are listed for each of the three themes.

#### Theme: Support for Cycle Syncing Is Influenced by a Patriarchal System

##### Referent: Men

Participants claimed that societal structures, including work and social forces, are greater aligned with male bodies. There is a perception that men, working on shorter daily cycles, may struggle to understand the nuances of women’s longer menstrual cycles, potentially hindering support for cycle syncing.

I feel like also in terms of just like the society in general, we live in a patriarchal society. So that has influenced the timing of things and the way the timing of our days or even weeks and months are planned out, like just as a society is aligned to a man cycle and not a woman cycle. As a society, men would never understand that we need more rest.

Yeah, I hate to say this, but I feel like men could be like a little more hesitant with like backing the idea, just because I know men work on like a 30-day cycle or something like that, or a 24-hour cycle. It’s like they wake up, and that’s their day and then it’s like starts over for them. But I know women are like 30 days. I think like they [men] wouldn’t maybe understand symptoms like fatigue. But if I was working with a team of women, they’d give me more grace if I was on my period.

##### Referent: Brothers

As a result of societal forces and structures, participants felt like the men in their lives wouldn’t be supportive of cycle syncing.

My brothers, just because they’re guys, they don’t really understand it. I don’t have any sisters. So like I’m the only girl in my family. But if I like said told my brothers what I was doing, they might be like, what are you doing? Like why? Just like live with the pain.

#### Theme: Generational Support for Cycle Syncing Varies

##### Referent: Older Generation

Referent’s normative assumptions about cycle syncing vary by generation. Some participants suggested that older generations may dismiss the practice, viewing it as unnecessary: “The older generations who haven’t done this and have like, been fine without it may just have that like ideal of like, I didn’t need this and I was fine. It’s just your generation like making something up.”

##### Referent: Younger Friends

In contrast, there is optimism among some participants that friends closer in age are likely to support cycle syncing as they share a more open and supportive attitude toward menstruation and reproductive health.

I would say probably my friends more than anything because I think that like some of our parents took over a generation of like talking about periods is gross and like don’t talk about that around your dad. My friends … of the same generation [are] real and very open about our bodies and if something that I thought would benefit my health and my mental health, I think they would be very supportive of my decision.

#### Theme: Female Solidarity in Practices That Involve Reproductive Care

##### Referent: Women

Participants indicated a strong sense of female solidarity, with participants expressing confidence that their mothers and sisters would be enthusiastic and supportive of these practices. This reflects the value placed on intergenerational wisdom and a shared commitment to well-being within families. For example, many participants described support for cycle syncing and reproductive self-care practices among the women in their lives: “Most of the women in my life may support it because I feel like at least the people I surround myself with they’re pretty open-minded.”

##### Referent: Mothers

Another group of participants focused primarily on the support of their mothers and sisters:

My mom would definitely feel that way [supportive]. She is very much like a health freak like work out like, eat all the right stuff all the time, and like II never hear the end of it at home, so in general, I’m sure if she’s not already familiar to this, I’m sure if she was that even herself, she would follow like everything to do with it.

##### Referent: Sisters

Like mothers, participants discussed the support they might receive from their sisters: “I think my sisters would like that cause like I just like, I said, I get high and low, so I feel like, if I like tracked it, it would like help my mood a lot. So I think they would want me to do that.”

##### Referent: Friends

Participants highlighted the influence of their friends and roommates, viewing the idea of group activities and mutual support as a means to enhance their relationships and ensure collective accountability.

It might be something that could be like a group activity kind of do together, like eating certain foods and like exercising together. It could be kind of like a thing that you can bring people together and kind of just like, support each other and like doing something new or trying something.

### RQ3: What Barriers and Facilitators Do Young Women Mention in Relation to Cycle Syncing?

Participants listed several things that would make it easy and difficult to cycle sync. Two themes emerged: the significance of social support is key in achieving reproductive health goals and accessibility, women’s health education, and bodily autonomy. For each of the two themes, we list specific barriers and facilitators to cycle syncing.

#### Theme: Social Support is Key in Achieving Reproductive Health Goals

##### Facilitator: Social Support

This theme emphasizes the importance of collective support systems, especially when navigating issues related to reproductive health. For example, many participants suggested that cycle syncing would be more feasible if they had “An accountability buddy. So doing this with a friend. Social support.” Participants also highlighted how encouragement and accountability from friends and peers play a pivotal role in achieving goals, even if these supporters aren’t directly involved with cycle syncing.

I think like the most important part would be everybody around you also supporting it and like not necessarily that they have to do it with you, but just like, for example, like I make dinner for my roommates, like me, my roommates. So like, that would affect them as well.

#### Theme: Accessibility, Women’s Health Education, and Bodily Autonomy

##### Barrier: Knowledge

Participants emphasized the need for comprehensive and reliable information about women’s bodies and health. Specifically, they expressed a gap in traditional health education and women’s menstrual cycle phases, which would serve as a barrier to performing potentially beneficial reproductive health behaviors: “I feel like a concept that everyone is like kind of alluding to is like education … I can definitely tell you, I didn’t learn about cervical mucus and middle school health education.” Similarly, others said, “I think a good understanding of the different phases would be very helpful. Cause then you can also implement that into how your body reacts in those phases.” Others were uncertain about following the recommendations for and implementing exercise routines: “I have no clue what that is like for exercise, and a lot of the time people will walk into a gym and they have no idea what they’re doing.”

##### Barrier: Time

Time constraints, especially for busy individuals like college students, emerged as a significant barrier to implementing cycle syncing.

In an ideal world like if you didn’t have work, or whatever you know, kids ... If you have like a lot of things going on, you have a busy day like you said. It’s hard to make sure you know right things, squeezing time for extra exercise and get everything else you have to get done. So I think, just like you said, time.

##### Barrier: Accessing Healthy Food

Accessibility issues extended beyond information to include challenges in obtaining suitable foods and navigating exercise routines. For example, some participants struggled with finding recommended food for the menstrual phases: “The concept of the accessibility to the foods could be an issue.”

##### Barrier: Using Birth Control

Cycle syncing faces challenges in individuals with irregular menstrual cycles or those utilizing birth control methods. Participants expressed difficulties in tracking and aligning with specific phases due to the absence of regular cycles.

Yeah, I also don’t get my period because I have an IUD so like. But before, like I got one like, I would like, have all those cycles, and like. I think sometimes I notice like more bloating, or I’m more like on edge with things. So it’s kinda hard like for me, at least like to track that if I don’t really get my period or get any of those like cycles really.

## Discussion

The purpose of this study was to understand beliefs pertaining to attitudes, norms, and control in the context of cycle syncing. Cycle syncing is a newly emerging behavior on social media that has important implications for the future of women’s health. Through focus group discussions, three key findings emerged from the study. What follows is a discussion of these three key findings, as well as theoretical implications and future directions.

The first key finding suggests that cycle syncing is part of a larger discussion in the medical world about being natural and resisting medicalization. The theme of resisting medicalization revealed that participants saw cycle syncing as beneficial for reducing negative symptoms associated with menstruation and treating menstrual health naturally. In line with CFT, cycle syncing represents an attempt to reclaim agency over one’s body and health practices, resisting the dominant medical paradigm that often seeks to control or suppress natural physiological processes. However, the focus on being natural is consistent with previous research on social media and reproductive health which found that social media influencers encourage the discontinuation of hormonal contraception to be more natural (Pfender & Devlin, 2023) and avoid side effects (Pfender, Tsiandoulas, et al., 2024; Pfender, Wanzer, et al., 2024). A focus on resisting medicalization echoes recent work on “hormonophobia” (Foran, 2019) and negative discourse about the use of hormonal contraception (Pfender & Fowler, 2024; Schneider-Kamp & Takhar, 2023). For example, participants described the use of hormonal contraception as a barrier to cycle syncing because it can prevent menstruation, and a regular menstrual cycle is necessary for engaging in cycle syncing. Consequently, cycle syncing content could further fuel negative anti-hormonal contraceptive discourse on social media, a concern for unintended pregnancy and uptake of Opill, the first over-the-counter birth control pill.

The second key finding suggests that despite inconclusive evidence for cycle syncing in the scientific literature (Carmichael et al., 2021), there is strong support for and positive attitudes toward cycle syncing. For example, the elicitation revealed several positive attitudinal beliefs, such as cycle syncing can facilitate self-acceptance and provide symptom relief. Additionally, normative beliefs among women were prevalent, with many participants suggesting that mothers, sisters, and friends would likely support engagement with this health behavior. This is particularly concerning as much of the cycle syncing content disseminated on social media platforms is piecemeal (Pfender, Wanzer, et al., 2024), drawn from fragmented interpretations of the scientific literature rather than a comprehensive and nuanced understanding. Based on previous content analysis research, the fact that cycle syncing evidence is inclusive isn’t articulated on social media (Pfender, Wanzer, et al., 2024). Misinterpretation or oversimplification of research findings may lead to exacerbating the dissemination of misinformation (Sunkara, 2021). Consequently, individuals may be misled into adopting cycle syncing practices without a clear understanding of the efficacy or potential risks, highlighting the importance of promoting evidence-based information and critical appraisal of wellness trends in digital spaces.

The third key finding highlights a gendered element regarding support for cycle syncing and women’s health in that participants consistently listed men as people who would not support cycle syncing. They identified that skepticism or lack of support from men in their lives, such as brothers and fathers, could dissuade women from engaging in cycle syncing. Women might internalize these perceived attitudes and alter their behavior accordingly, either by downplaying the importance of menstrual health or feeling hesitant to openly discuss their health needs. This finding highlights CFT’s premise that patriarchal systems often shape health norms and perceptions, contributing to a broader pattern of women navigating their health choices within the context of societal expectations and male-centric perspectives.

Perceived male resistance also sheds light on deeper societal attitudes and potential policy implications. The notion that men may resist or lack understanding of cycle syncing implies a broader challenge in achieving gender-inclusive health policies. The identification of men as unsupportive stakeholders suggests that decision-making processes related to women’s health, including menstrual and reproductive matters, may be influenced by a male-centric perspective (Dayi, 2005). This finding aligns with other women’s health initiatives, such as Spain’s menstrual leave bill, passed in February 2023, which allows those with a documented history of painful periods to take paid menstrual leave from work. Though there is widespread support for the menstrual leave bill, others worry that the new legislation will further stigmatize women or result in negative consequences in the workplace, such as not getting promoted at the same rate as men (Le Monde, 2023).

Gendered dynamics also align with concerns raised in the literature about male doctors or figures dismissing women’s pain in healthcare settings, reflecting a systematic issue (Becker & McGregor, 2017; Samulowitz et al., 2018). Yet, participants did not name medical doctors as people who might support cycle syncing. This finding underscores the importance of addressing gender biases in healthcare policy formulation and implementation, advocating for more inclusive and empathetic approaches that consider the diverse needs and experiences of all individuals (McGlothen-Bell et al., 2023). In doing so, there is an opportunity to bridge the gap in understanding between genders and foster a more supportive and inclusive environment for women’s health behaviors and initiatives.

To comprehensively understand cycle syncing beliefs and their implications for reproductive health behaviors, it is essential to integrate both traditionally quantitative methods with critical theories. While the RAA provides valuable insights through quantitative measures of determinants and predictors of health behaviors, it is equally important to incorporate CFT to explore the underlying power dynamics and normative expectations that shape these behaviors. Cycle syncing is influenced by societal expectations and gendered norms, which impact perceptions of what is considered acceptable or natural. By combining the RAA’s quantitative approach with CFT’s critical lens, we can delve deeper into how social media trends reflect and reinforce these power structures and normative influences.

### Limitations and Future Directions

There are several limitations within the current study that serve as directions for future research. First, participants identified primarily as White or Latinx. Future research should investigate cycle syncing behaviors with a more diverse sample. Additionally, the information obtained from the focus groups, while useful, is not generalizable. Future research should use these data on attitudes, norms, and control to develop quantitative surveys to better understand which reasoned action actors predict the uptake of cycle syncing behavior. Finally, an important limitation of this research is the lack of research on cycle syncing itself. It is unclear whether this health behavior is advantageous to women’s health or just another social media health trend (Pfender, Wanzer, et al., 2024). Medical interventions should be conducted to understand if cycle syncing is a beneficial health behavior.

## Conclusion

This study investigated the emerging health behavior of cycle syncing on social media, aiming to uncover key beliefs regarding attitudes, norms, and control. The findings highlighted a connection between cycle syncing and “hormonophobia,” exposing negative discourse around hormonal contraception that may exacerbate anti-hormonal contraceptive sentiments on social media. Second, the study highlighted the acceptance of cycle syncing among women, despite inconclusive clinical evidence. Third, perceived male resistance was mentioned as a potentially influencing individual-level behavior among women, signaling broader societal attitudes and advocating for more inclusive healthcare policies. Theoretical implications underscore the need for a mixed methods approach, integrating critical theories to dissect power dynamics and normative influences in health behaviors. Future research should use quantitative methods to understand the effects of cycle syncing health messaging and medical interventions to discern the actual benefits of cycle syncing for women’s health.
